# Combining visual rating scales to identify prodromal Alzheimer's disease and Alzheimer's disease dementia in a population from a low and middle-income country

**DOI:** 10.3389/fneur.2022.962192

**Published:** 2022-09-01

**Authors:** Nilton Custodio, Marco Malaga, Diego Chambergo-Michilot, Rosa Montesinos, Elizabeth Moron, Miguel A. Vences, José Carlos Huilca, David Lira, Virgilio E. Failoc-Rojas, Monica M. Diaz

**Affiliations:** ^1^Servicio de Neurología, Instituto Peruano de Neurociencias, Lima, Peru; ^2^Unidad de diagnóstico de deterioro cognitivo y prevención de demencia, Instituto Peruano de Neurociencias, Lima, Peru; ^3^Unidad de Investigación, Instituto Peruano de Neurociencias, Lima, Peru; ^4^Escuela Profesional de Medicina Humana, Universidad Privada San Juan Bautista, Lima, Peru; ^5^San Martin de Porres University, Lima, Peru; ^6^Universidad Científica del Sur, Lima, Peru; ^7^Departamento de Radiología, Hospital Nacional Edgardo Rebagliati Martins, EsSalud, Lima, Peru; ^8^Servicio de Radiología, Centro de Diagnóstico por Imagen-DPI, Lima, Peru; ^9^Departamento de Neurología, Hospital Nacional Edgardo Rebagliati Martins, EsSalud, Lima, Peru; ^10^Servicio de Neurología, Hospital Guillermo Kaelin de La Fuente, Lima, Peru; ^11^Centro de Investigación en Medicina Traslacional, Universidad Privada Norbert Wiener, Lima, Peru; ^12^Department of Neurology, University of North Carolina at Chapel Hill, Chapel Hill, NC, United States; ^13^Facultad de Salud Pública y Administración, Universidad Peruana Cayetano Heredia, Lima, Peru

**Keywords:** Alzheimer's disease, mild cognitive impairment, magnetic resonance imaging, visual rating scores, medial temporal atrophy score

## Abstract

**Background:**

Many low- and middle-income countries, including Latin America, lack access to biomarkers for the diagnosis of prodromal Alzheimer's Disease (AD; mild cognitive impairment due to AD) and AD dementia. MRI visual rating scales may serve as an ancillary diagnostic tool for identifying prodromal AD or AD in Latin America. We investigated the ability of brain MRI visual rating scales to distinguish between cognitively healthy controls, prodromal AD and AD.

**Methods:**

A cross-sectional study was conducted from a multidisciplinary neurology clinic in Lima, Peru using neuropsychological assessments, brain MRI and cerebrospinal fluid amyloid and tau levels. Medial temporal lobe atrophy (MTA), posterior atrophy (PA), white matter hyperintensity (WMH), and MTA+PA composite MRI scores were compared. Sensitivity, specificity, and area under the curve (AUC) were determined.

**Results:**

Fifty-three patients with prodromal AD, 69 with AD, and 63 cognitively healthy elderly individuals were enrolled. The median age was 75 (8) and 42.7% were men. Neither sex, mean age, nor years of education were significantly different between groups. The MTA was higher in patients with AD (*p* < 0.0001) compared with prodromal AD and controls, and MTA scores adjusted by age range (*p* < 0.0001) and PA scores (*p* < 0.0001) were each significantly associated with AD diagnosis (*p* < 0.0001) but not the WMH score (p=0.426). The MTA had better performance among ages <75 years (AUC 0.90 [0.85–0.95]), while adjusted MTA+PA scores performed better among ages>75 years (AUC 0.85 [0.79–0.92]). For AD diagnosis, MTA+PA had the best performance (AUC 1.00) for all age groups.

**Conclusions:**

Combining MTA and PA scores demonstrates greater discriminative ability to differentiate controls from prodromal AD and AD, highlighting the diagnostic value of visual rating scales in daily clinical practice, particularly in Latin America where access to advanced neuroimaging and CSF biomarkers is limited in the clinical setting.

## Introduction

The prevalence of dementia in Latin America and the Caribbean (LAC) is high compared to that of high-income countries and is expected to triple by the year 2050 (1). This increase is expected to occur rapidly in populations with low educational levels and illiteracy (2) due to increased life expectancy and improved health outcomes over time (3). Alzheimer's disease (AD) is one of the most common diseases of old age. In LAC, a genetic contribution to AD may play a significant role given single mutations associated with altered amyloid metabolism have been reported in various familial cases of AD throughout LAC (4, 5). The diagnosis of AD has evolved over time and is now defined using biomarkers that allow for categorization into different pre-dementia and dementia stages (6). However, many of these biomarkers are scarce in LAC (7). Although the majority of LAC countries have access to brain magnetic resonance imaging (MRI) to aid in the diagnosis of AD, few cities in LAC have access to brain imaging using positron emission tomography (PET) with amyloid or tau tracers (2), recommended in the diagnostic work-up of AD (6). Moreover, although amyloid and tau CSF biomarkers, amyloid and tau brain PET, and genetic testing for APOE genotype may be available in Latin America, these diagnostic modalities are limited to research settings and are rarely available in the clinical setting, increasing the difficulty in appropriate diagnosis of prodromal AD and AD.

Other imaging modalities are limited to the research setting, but are not available in the clinical setting in LAC. Functional MRI (fMRI), for example, is utilized to investigate cognitive impairment in many research settings. However, in LAC, the most widely available clinical methods to assess relationships between brain structures and their functions are neuropsychological testing and visual assessments of cortical atrophy patterns on brain MRI imaging by the practitioner (8). The most widely used MRI visual scoring system is the medial temporal lobe atrophy (MTA) score (9), which has a high sensitivity for the detection of prodromal AD (or mild cognitive impairment due to AD) and AD (10). Other MRI visual rating scales validated for use for dementia include the posterior atrophy (PA) scale (11) and global cortical atrophy-frontal (GCA-F) scale, which may serve as potential biomarkers for atypical AD and non-AD dementias (8). White matter lesions or white matter hyperintensities (WMH) may also be associated with cognitive decline depending on the lesion burden and location. Fazeka's scale is the most widely used visual rating scale used to characterize WMH burden (12) and may help characterize non-AD dementias. In LAC, Brazil and Argentina are the leaders in neuro-imaging research in dementia (13) with resources available to implement the standardized MRI AD evaluation protocol established by the Alzheimer's Disease Neuroimaging Initiative (ADNI) (14). Most Latin American countries, including Peru, lack medical centers with the capacity to diagnose dementia using amyloid and tau cerebrospinal fluid (CSF) biomarkers, neuropsychological testing, and standardized ADNI MRI protocols. Therefore, training general medical personnel in the interpretation of MRI visual radiological scales may serve as an alternative that may aid in the diagnosis of prodromal AD and AD in low and-middle-income countries that may not have access to those recommended diagnostic modalities (2). For these reason, the aim of the present study was to evaluate the diagnostic performance of the combination of visual assessment scales to identify prodromal AD and AD.

## Materials and methods

### Design and participants

A cross-sectional, prospective study was conducted to quantify the degree of cortical atrophy identified by visual scoring systems in MRIs of the brain of cognitively healthy controls compared with patients with prodromal AD and AD.

**Figure 1:**
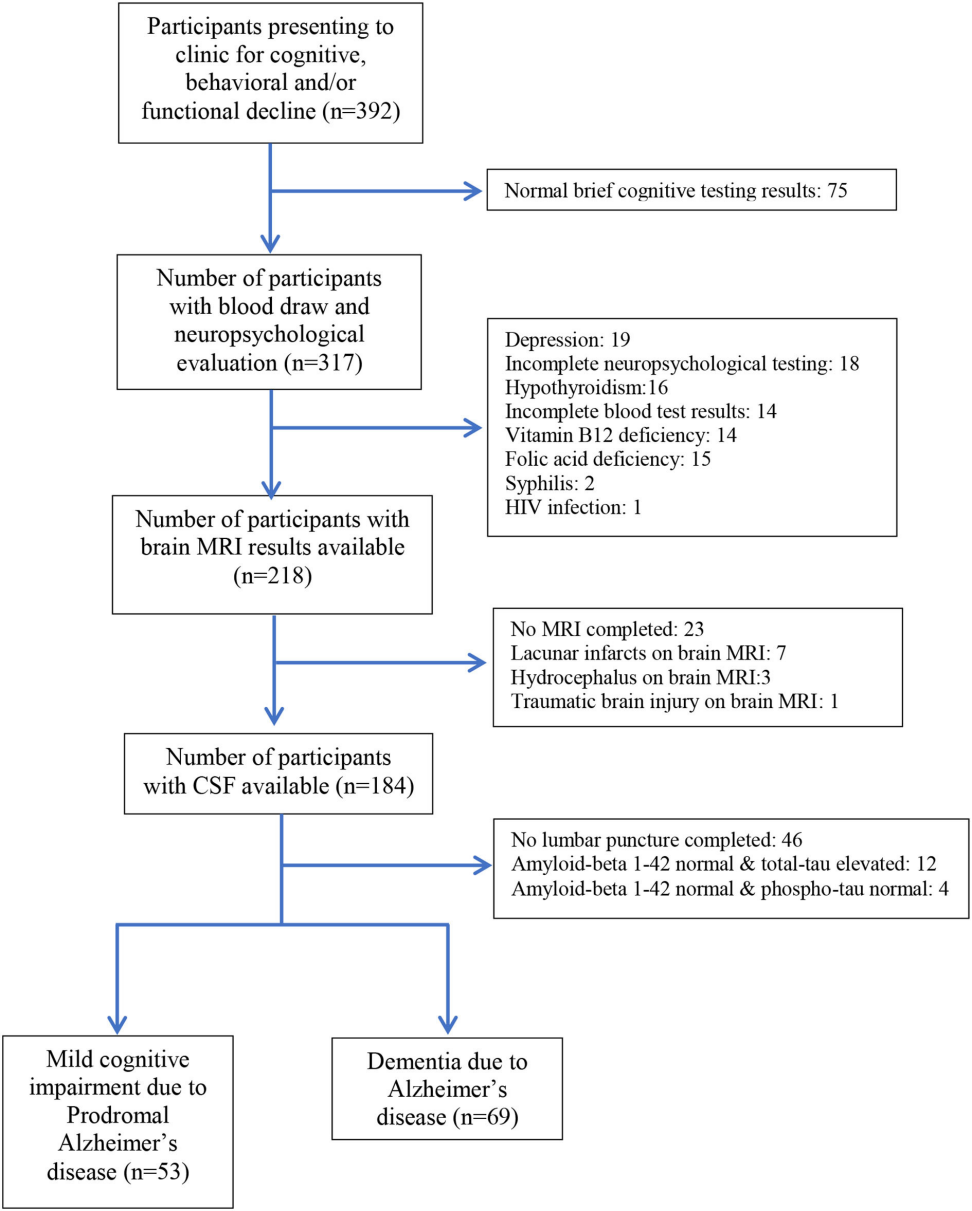
Participant selection flowchart for patients with Alzheimer's disease and prodromal Alzheimer's disease.

We enrolled patients from the Neurology service and Cognitive Impairment Diagnosis and Dementia Prevention Unit of the *Instituto Peruano de Neurociencias* (Peruvian Neurological Institute; IPN) located in Lima, Peru between January 2019 and October 2021. Eligible patients with prodromal AD, AD, and cognitively healthy controls were invited to participate (Figure 1). Exclusion criteria included the following: prior history of head trauma resulting in loss of consciousness, active epilepsy, prior history of stroke, or a person unable to undergo MRI due to metal in the body or severe claustrophobia. In addition, we excluded participants with any abnormal findings on MRI that would suggest prior lacunar ischemic infarcts, brain tumors, traumatic brain injury, or other pathology on neuroimaging deemed by the investigators to confound the results of neurocognitive testing or the MRI visual rating scale scores.

All participants underwent neuropsychological assessment (brief cognitive tools screening and standardized neuropsychological battery), dementia risk factor questionnaires, a lumbar puncture for CSF amyloid and tau levels, and a brain MRI utilizing the standardized protocol defined by the Center for Radiology, *Diagnóstico por Imágenes* (DPI in Spanish) in Lima, Peru. The diagnosis of prodromal AD was made utilizing the minor neurocognitive disorders criteria from the Diagnostic and Statistical Manual of Mental Disorders-5 (DSM-5) criteria of MCI (15). Cognitive impairment was classified using the patient's cognitive profile (cognitive domain impairment >1.5 SD below age- and gender-appropriate norms on neuropsychological tests). The study neuropsychologist further categorized participants with MCI into amnestic (prodromal AD) and non-amnestic MCI based on baseline neuropsychological tests. Scores greater than −1.5 SD from the mean compared to norms on the verbal and/or visual episodic memory domain tasks were classified as prodromal AD. Normal scores in memory domains combined with scores of more than −1.5 SD from the mean in one or more of the other domains assessed was categorized as non-amnestic MCI (16). The AD group consisted of patients with a diagnosis of typical AD according to the published criteria from McKhann et al. (17). Once patients were classified based on neuropsychological testing as either prodromal AD, non-amnestic MCI, or AD, only those that were considered prodromal AD or AD went on to have a lumbar puncture. Prodromal AD and AD diagnosis were confirmed if a patient demonstrated high tau and low amyloid levels in CSF. Only these patients were entered in our analyses. A control group of 63 cognitively healthy volunteers were recruited from local newspapers, radio, and social media. All controls had normal scores on neuropsychological testing and normal CSF amyloid and tau levels. These controls were matched to the prodromal AD group by age and sex.

### Neuropsychological assessments

The following neuropsychological tests were administered to all participants.

#### Rowland Universal Dementia Assessment Scale

The RUDAS is a simple tool administered within 10 min and comprised of 6 components (memory, visuospatial orientation, visuospatial praxis, motor praxis, judgment, and language). The RUDAS has a maximum score of 30, where a lower score denotes poor cognitive performance (18). Several studies have been published validating the RUDAS among Peruvians with a middle-education level from an urban area of Peru (19) and Peruvians with illiteracy (20).

#### Memory alteration test

The Memory alteration test (M@T) is a valid screening test that assesses various memory types (episodic, contextual, and semantic) and discriminates between healthy elderly subjects, patients with prodromal AD, and those with early AD. The test was developed in Spain and has high internal consistency and validity, brief testing time (5–10 min), and is easy to perform and interpret (21). Moreover, the test has been validated among Peruvians with middle-educational levels (22) and for older adults with low educational levels (22). The M@T appropriately discriminates between cognitively healthy status, MCI, and AD (22).

#### Neuropsychological battery

The neuropsychological battery consisted of the following tests: Rey Auditory Verbal Learning Test, Logical Memory Subtest of the revised Weschler Memory Scale, Trail Making Tests A and B, Rey Complex Figure, Boston Naming Test, Wisconsin Card Sorting Test, Letter-Number (subtest of the Weschler Adult Intelligent Scale III), Digit Span, Strub-Black Picture Copying, and the WAIS-III Cubes Test, as has previously been described (22). This battery was administered by a licensed study neuropsychologist.

### MRI of the brain

#### Data acquisition

Images were acquired using a 3 Tesla Siemens Skyra MR System. Study participants underwent an MRI using a standardized protocol containing volumetric T1-weighted magnetization-prepared rapid gradient echo (MP-RAGE) and fluid attenuation inversion recovery (FLAIR) sequences. Following a pilot scan, 3 Plane/Tri-Planar Scout/Calibration Scan, whole-brain sagittal structural T1-rapid gradient-echo, 3D FLAIR sagittal, 3D arterial spin labeling (ASL), coronal T2, and Accelerated High-Resolution Hippocampus Scan (oblique acquisition with 2 mm thick slices perpendicular to the long axis of the hippocampi) were performed. The total scan time was 25 min.

#### Scoring systems

Visual radiological scoring systems were used to assess brain pathology in patients with prodromal AD, AD, and in controls, including the Schelten's Medial Temporal Lobe Atrophy (MTA) score, Fazekas's scale to measure WMH burden, and the PA score. We evaluated the MTA and PA using T1-weighted images and the Fazeka's score using FLAIR images. To score each scale, an experienced radiologist (EM-C) and neurologist (NC) viewed the images independently at separate locations, and both were blinded to group allocation. Reference images for all scores were provided for both the radiologist and neurologist as suggested by Harper et al. (23). A consensus rating was held if a disagreement existed between the two raters. For all scores except the Fazekas and PA scores, both brain hemispheres were scored and a mean score was calculated. The mean score was calculated based on both brain hemispheres for the MTA (24, 25).

The MTA score ranges from 0 to 4 (0 = no atrophy to 4 = most severe atrophy) and describes the relative size of the hippocampus at a fixed position on T1 sequences. The MTA score cut-offs were set at 1.0 for persons under 65, 1.5 for persons between 66 and 74 years of age, and 2 for those 75 years or older, as has been previously described (8, 26).

The PA scoring system ranges from 0 to 3 (0 = no atrophy, 1 = mild, 2 = moderate, and 3 = severe atrophy). The original age cut-offs previously described for the PA scale were used (11). Fazeka's score quantifies nonspecific WMH burden using scores ranging from 1 to 3 (ranging from absent to greater WMH load depending on the location of the hyperintensities). A score >1 was considered pathological for all age groups (27). For all radiological scoring systems, scores above the set cut-off values were considered pathological.

Lastly, we included two new scores developed by adding the mean MTA or the age-adjusted MTA scores (using the established cut-off scores by age range for the MTA; 1.0: <65 years, 1.5: 66–74 years, 2: ≥ 75 years) to the PA score, respectively. The cut-off score was determined by receiver–operator curves as the point with the highest percentage of correctly classified patients.

### CSF biomarker analysis

Cerebrospinal fluid was obtained through lumbar puncture performed and tested for amyloid-beta 1-42, phospho-tau, and total-tau using commercial ELISA test kits.

### Statistical analysis

Demographic and cognitive characteristics of the population were described using measures of central tendency and dispersion for continuous variables and frequencies for categorical variables. We compared these characteristics among the control, prodromal AD, and AD groups to ensure there were no significant differences that could potentially confound results. Additionally, we compared CSF biomarker values among patients with different visual radiological scores using a non-paired t-test. Next, we compared the MTA and adjusted MTA mean scores between diagnostic groups, as well as the PA and WMH Fazekas's score frequencies. We used the ANOVA and Chi-square tests for means and frequencies, respectively.

Lastly, we assessed the sensitivity, specificity, and area-under-the-curve (AUC) with 95% confidence intervals (CI) for each score, outcome, and age group using the ROC analysis. We divided patients into two age groups: under 75 and 75 and older. Additionally, we explored three outcomes: diagnosis of prodromal AD, prodromal AD or AD, and a diagnosis of AD only.

For scores without pre-established cut-off scores, we reported the sensitivity and specificity at the point with the highest percentage of correctly classified patients. For the MTA+PA score, the cutoff score was determined at 2.5 for both prodromal AD alone and prodromal AD or AD combined, and at 3.5 for AD only. For adjusted MTA+PA scores (adjusted based on the established cut-off score for each age range), the cut-off score was 2 for all outcomes. STATA v16 software (Texas, USA) was used for all statistical analyses.

### Ethical considerations

The research activities involved in this study have been conducted in accordance with the ethical standards of the Helsinki Declaration. The study was approved by the Committee for medical and health research ethics, Hospital Nacional Docente Madre-Niño-HONADOMANI “San Bartolomé” (no: 10777-18). All participants participated voluntarily in the study and provided written informed consent.

## Results

We enrolled a total of 185 patients: 63 controls, 53 patients with prodromal AD, and 69 with AD. We found that 42.7% were men and the median age was 75 (interquartile range [IQR] 70–78). Most participants had more than 12 years of education. Neither sex, age, nor years of education were significantly different between groups. As expected, the median scores for the CDR and RUDAS were significantly higher among the participants with prodromal AD and AD and were significantly lower for M@T scores compared with healthy controls (Table 1).

**Table 1:** Demographic characteristics and different imaging scores for patients with AD, MCI, or controls.

	**Diagnosis N(%)**				***p***
	**Control (*n =* 63)**	**Prodromal AD (*n =* 53)**	**AD (*n =* 69)**	**Total (*N =* 185)**	
**Sex**					0.060
Female	43 (68.3)	30 (56.6)	33 (47.8)	106 (57.3)	
Male	20 (31.8)	23 (43.4)	36 (52.2)	79 (42.7)	
Age**	76 (7)	75 (7)	74 (9)	75 (8)	0.419
Years of education*	12.2 (3)	12.1 (2)	11.9 (2)	12.1 (2)	0.791
CDR**	0 (0)	0.5 (0)	1 (0)	0.5 (1)	0.000
M@T**	45 (3)	34 (3)	18 (4)	34 (23)	0.000
RUDAS**	26 (2)	22 (2)	19 (4)	22 (6)	0.000
MTA***	1.2 (1)	1.9 (0)	2.8 (0)	2.0 (1)	0.000
MTA***			0.000
No	44 (69.8)	10 (18.9)	0 (0.0)	54 (29.2)	
Yes	19 (30.2)	43 (81.1)	69 (100.0)	131 (70.8)	
**WMH**					0.426
0	23 (36.5)	16 (30.2)	20 (29.0)	59 (31.9)	
1	32 (50.8)	23 (43.4)	29 (42.0)	84 (45.4)	
2	7 (11.1)	11 (20.8)	15 (21.7)	33 (17.8)	
3	1 (1.6)	3 (5.7)	5 (7.3)	9 (4.9)	
**Parietal atrophy**					0.000
0	40 (63.5)	17 (32.1)	0 (0.0)	57 (30.8)	
1	18 (28.6)	30 (56.6)	3 (4.4)	51 (27.6)	
2	5 (7.9)	6 (11.3)	41 (59.4)	52 (28.1)	
3	0 (0.0)	0 (0.0)	25 (36.2)	25 (13.5)	

*Mean (SD) ^**^Median (IQR).

***MTA scores based on previously established cut-offs for various age ranges (1.0: <65 years, 1.5: 66-74 years, 2: >=75 years). CDR, Clinical Dementia Rating; M@T, Memory Alteration Test; MTA, Medial Temporal Lobe Atrophy scale; PA, Posterior Atrophy scale; RUDAS, Rowland Universal Dementia Assessment Scale; WMH, Fazeka's white matter hyperintensity scale.

For the visual rating scores, we found that the MTA was significantly higher in patients with AD (*p* < 0.001) compared with MCI and controls using bivariate analyses, and adjusted MTA was significantly associated with AD diagnosis (*p* < 0.001). The same was true for the PA score (*p* < 0.001), but not for the WMH score (*p* = 0.426).

We then evaluated the sensitivity, specificity, and AUC of different visual rating scores, for the diagnoses of prodromal AD/AD, prodromal AD only, and AD only using three groups: all patients, patients 75 years or younger, and those older than 75. We included all previously mentioned scores and two composite scores: MTA+PA and adjusted-MTA+PA scores.

**Figure 2:**
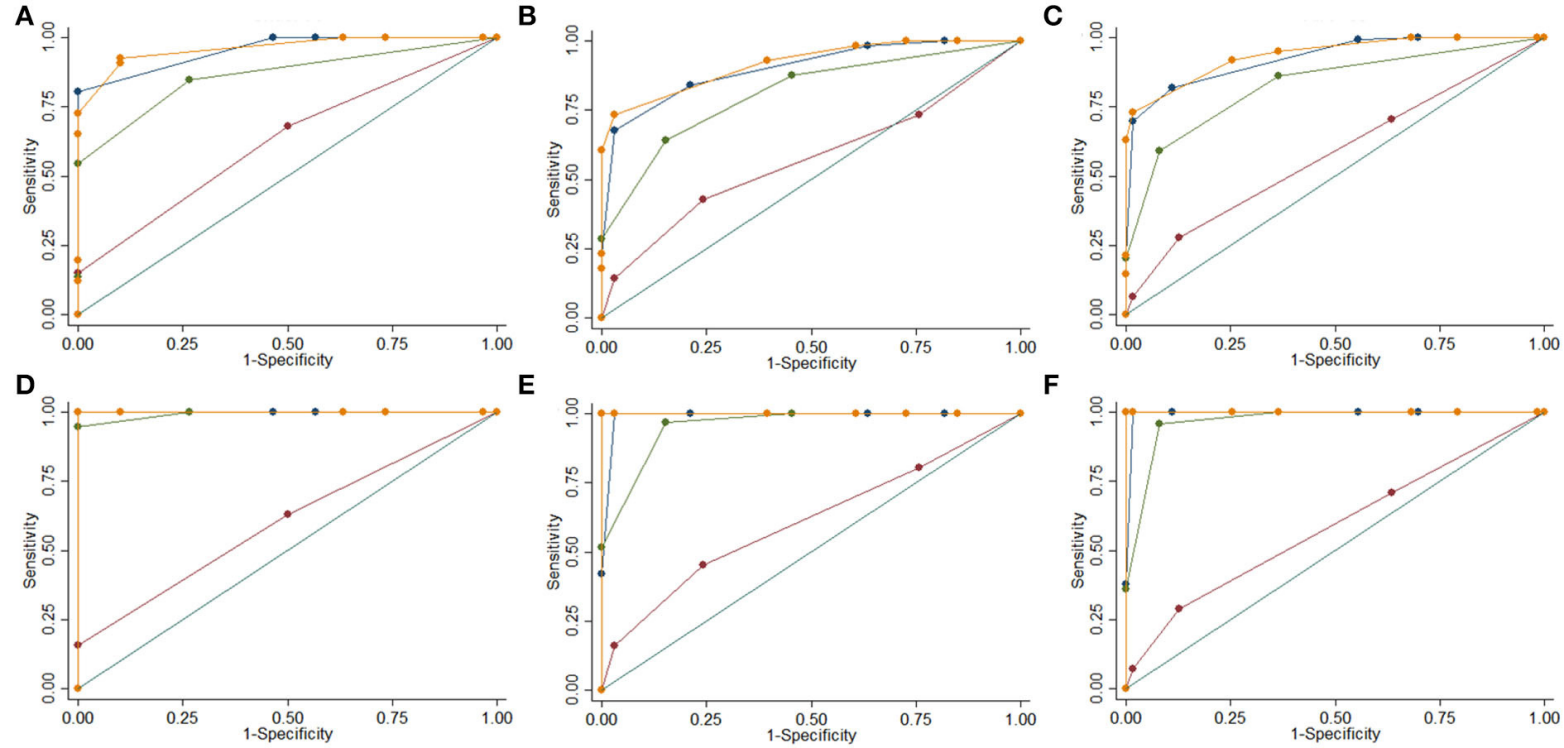
ROC Curves for different MRI visual rating scales. MTA Non-Adjusted (Blue), WMH (Brown), PA (Green), MTA + PA (Orange). Control vs. prodromal AD or AD 75 or under 75 **(A)**, over 75 **(B)**, all ages **(C)**; Control vs. AD 75 or under 75 **(D)**, over 75 **(E)**, all ages **(F)**.

For the diagnosis of prodromal AD/AD, the MTA, MTA+PA, and adjusted-MTA+PA had the best performance with an AUC of 0.85 (0.80–0.91), 0.87 (0.82–0.91), and 0.83 (0.77–0.89) respectively. Out of these, MTA had better performance among patients 75 years or younger (AUC 0.90 [0.85–0.95]), and the MTA+PA had better performance in patients older than 75 (AUC 0.85 [0.79–0.92]) (Figures 2A–C).

For prodromal AD alone, both adjusted MTA and adjusted MTA+PA had AUCs >0.75 (0.76 [0.68–0.83] and 0.78 [0.70–0.86]), respectively. Both unadjusted MTA and MTA+PA AUC's were close at 0.74 (0.66–0.81) and 0.73 (0.66–0.81), respectively. For patients 75 years or younger, age-adjusted MTA+PA was significantly better at identifying prodromal AD (AUC 0.84 [0.75–0.94]). However, for patients >75 years of age all scores had an AUC <0.75, and adjusted MTA+PA had the best performance (AUC 0.72 [0.61–0.84]) for identifying prodromal AD.

For AD alone, the diagnostic performance of the MTA and both MTA+PA was high with an AUC of 0.99 for all patients (Figures 2D–F). However, MTA+PA had the best performance (AUC 1.00; 100% sensitivity and specificity) for all three groups. Lastly, out of all scores, the WMH demonstrated the lowest performance with AUCs of 0.5 for all age groups and outcomes (prodromal AD, prodromal AD or AD, and AD alone) (Table 2).

**Table 2:** Sensitivity, specificity, and AUC for different imaging scoring methods for diagnosis of prodromal AD or AD, or AD dementia only.

**Score**	**Control vs. prodromal AD + AD**			**Control vs. prodromal AD**			**Control vs. AD**		
	**Sensitivity**	**Specificity**	**AUC**	**Sensitivity**	**Specificity**	**AUC**	**Sensitivity**	**Specificity**	**AUC**
**MTA**	CUTPOINT ≥ 2						CUTPOINT ≥ 2.5		
≤ 75	80.3% (69–89)	100% (88–100)	0.90 (0.85–0.95)	53.6% (34–73)	100% (88–100)	0.77 (0.67–0.86)	100% (91–100)	100% (88–100)	1.00 (1.00–1.00)
>75	83.9% (72–92)	78.8% (61–91)	0.81 (0.73–0.90)	64.0% (43–82)	78.8% (61–91)	0.71 (0.60–0.83)	100% (89–100)	97.0% (84–100)	0.99 (0.96–1.00)
All	82.0% (74–88)	88.9% (78–95)	0.85 (0.80–0.91)	58.5% (44–72)	88.9% (78–95)	0.74 (0.66–0.81)	100% (95–100)	98.4% (92–100)	0.99 (0.98–1.00)
MTA**									
≤ 75	98.5% (92–100)	60.0% (41–77)	0.79 (0.70–0.88)	96.4% (82–100)	60.0% (41–77)	0.78 (0.69–0.88)	100% (91–100)	60.0% (41–77)	0.80 (0.71–0.89)
>75	83.9% (72–92)	78.8% (61–91)	0.81 (0.73–0.90)	64.0% (43–82)	78.8% (61–91)	0.71 (0.60–0.83)	100% (89–100)	78.8% (61–91)	0.89 (0.82–0.97)
All	91.8% (85–96)	69.8% (57–81)	0.81 (0.75–0.87)	81.1% (68–91)	69.8% (57–81)	0.76 (0.68–0.83)	100% (95–100)	69.8% (57–81)	0.85 (0.79–0.91)
**PA**									
≤ 75	84.8% (74–93)	73.3% (54–88)	0.79 (0.70–0.88)	64.3% (44–81)	73.3% (54–88)	0.69 (0.57–0.81)	100% (91–100)	73.3% (54–88)	0.87 (0.79–0.95)
>75	87.5% (76–95)	54.5% (36–72)	0.71 (0.61–0.81)	72.0% (51–88)	54.5% (36–72)	0.63 (0.51–0.76)	100% (89–100)	54.5% (36–72)	0.77 (0.69–0.86)
All	86.1% (79–92)	63.5% (50–75)	0.75 (0.68–0.82)	67.9% (54–80)	63.5% (50–75)	0.66 (0.57–0.74)	100% (95–100)	63.5% (50–75)	0.82 (0.76–0.88)
**FAZEKAS**									
≤ 75	68.2% (56–79)	50.0% (31–69)	0.59 (0.48–0.70)	75.0% (55–89)	50.0% (31–69)	0.63 (0.50–0.75)	63.2% (46–78)	50.0% (31–69)	0.57 (0.45–0.69)
>75	73.2% (60–84)	24.2% (11–42)	0.49 (0.39–0.58)	64.0% (43–82)	24.2% (11–42)	0.44 (0.32–0.56)	80.6% (63–93)	24.2% (11–42)	0.52 (0.42–0.63)
All	70.5% (62–78)	36.5% (25–50)	0.54 (0.46–0.61)	69.8% (56–82)	36.5% (25–50)	0.53 (0.45–0.62)	71.0% (59–81)	36.5% (25–50)	0.54 (0.46–0.62)
MTA**+ PA ≥2							
≤ 75	90.9% (81–97)	90.0% (74–98)	0.91 (0.84–0.97)	78.6% (59–92)	90.0% (74–98)	0.84 (0.75–0.94)	100% (91–100)	100% (88–100)	1.00 (1.00–1.00)
>75	92.9% (83–98)	60.6% (42–77)	0.77 (0.68–0.86)	84.0% (64–96)	60.6% (42–77)	0.72 (0.61–0.84)	100% (89–100)	100% (89–100)	1.00 (1.00–1.00)
All	91.8% (85–96)	74.6% (62–85)	0.83 (0.77–0.89)	81.1% (68–91)	74.6% (62–85)	0.78 (0.70–0.86)	100% (95–100)	100% (94–100)	1.00 (1.00–1.00)
**MTA + PA**	CUTPOINT ≥ 2.5						CUTPOINT ≥ 3.5		
≤ 75	84.8% (74–93)	90.0% (74–98)	0.87 (0.80–0.94)	64.3% (44–81)	90.0% (74–98)	0.77 (0.67–0.88)	100% (91–100)	90.0% (74–98)	0.95 (0.90–1.00)
>75	73.2% (60–84)	97.0% (84–100)	0.85 (0.79–0.92)	40.0% (21–61)	97.0% (84–100)	0.69 (0.58–0.79)	100% (89–100)	97.0% (84–100)	0.99 (0.96–1.00)
All	79.5% (71–86)	93.7% (85–98)	0.87 (0.82–0.91)	52.8% (39–67)	93.7% (85–98)	0.73 (0.66–0.81)	100% (95–100)	93.7% (85–98)	0.97 (0.94–1.00)

**MTA scores based on previously established cut-offs for various age ranges (1.0: <65 years, 1.5: 66-74 years, 2: >=75 years). CDR, Clinical Dementia Rating; M@T, Memory Alteration Test; MTA, Medial Temporal Lobe Atrophy scale; PA, Posterior Atrophy scale; RUDAS, Rowland Universal Dementia Assessment Scale; WMH, Fazeka's white matter hyperintensity scale. AD, Alzheimer's disease dementia.

Lastly, we compared different AD CSF biomarkers among patients with different MRI visual rating scale scores (Table 3). For all biomarkers (B-amyloid, t-tau, and p-tau), there was a statistically significant difference among patients with normal or pathological scores in age-adjusted MTA, WMH score, and parietal atrophy scores (*p* < 0.000). All biomarkers showed statistically significant correlation with MTA non-age adjusted values (p < 0.000).

**Table 3:** Correlation between MRI visual rating scale scores and Alzheimer's disease CSF biomarkers.

	**MTA**		MTA**– AD mean (SD)			**WMH mean (SD)**			**Parietal Atrophy mean (SD)**		
	**Corr Coef**	***p***	**Negative**	**Positive**	***p***	**Negative**	**Positive**	***p***	**Negative**	**Positive**	***p***
B-Amyloid	−0.005	0.000	414 (10)	294 (7.2)	0.000	364 (16)	355 (8.6)	0.5591	416 (14)	332 (8.3)	0.000
t-Tau	0.012	0.000	87 (1.9)	155 (4.6)	0.000	113 (5.8)	121 (4.2)	0.3275	84 (2.5)	134 (4.1)	0.000
p-Tau	0.021	0.000	49 (1.8)	82 (2.2)	0.000	63 (2.9)	65 (2.4)	0.6054	47 (2.4)	71 (2.13)	0.000

**MTA scores based on previously established cut-offs for various age ranges (1.0: <65 years, 1.5: 66-74 years, 2: >=75 years). AD, Alzheimer's disease; B-amyloid, beta-amyloid; Corr Coeff, correlation coefficient; MTA, Medial Temporal Lobe Atrophy scale; p-tau, phosphorylated tau; t-tau= total tau; PA, Posterior Atrophy scale; WMH, Fazeka's white matter hyperintensity scale.

## Discussion

In this study, we investigated the utility of brain MRI-based visual rating scales to identify patients with prodromal AD or AD in a resource-limited setting. These scales can be used as diagnostic biomarkers recommended by guidelines to diagnose AD (17). Our study found that among participants older than 75 years of age: (1) the MTA had acceptable discriminative properties to differentiate prodromal AD or AD from controls, but the MTA was better able to discriminate AD from controls; (2) the PA score alone had moderate strength in differentiating prodromal AD or AD from controls, improving considerably when applied to discriminate AD from controls; (3) the combination of the MTA and PA scores reach ideal levels for differentiating AD from controls.

The medial temporal lobe is a key anatomical structure of episodic memory, and structural alterations are found in most typical variants of AD, including prodromal AD and early stages of AD (28). Compared to more complex gray matter (GM) MRI volumetric analyses, the MTA visual rating scale is considered a brief measure that can be applied in daily clinical practice (10, 23, 29). For each age group and cutoff score, the MTA's AUC in our cohort demonstrated good discriminative properties for differentiating prodromal AD from controls with results similar to a cohort from one memory clinic in China (30), as well as for discriminating controls from AD (31). Furthermore, in a study of patients being followed longitudinally with prodromal AD who later converted to AD, these patients initially had GM volume loss at the level of the medial temporal lobe, including the hippocampus and entorhinal cortex (32). When compared to non-converting prodromal AD, converting prodromal AD showed a statistically significant degree of atrophy in the left hippocampus at baseline, suggesting that medial temporal lobe atrophy could be a topographic biomarker of conversion to AD in those patients with prodromal AD (30). Studies that prioritize sensitivity (rather than specificity) have been shown to increase MTA scores by 2 points to lower the risk of false positives. However, this carries an inadvertent disadvantage of also lowering the prodromal AD and AD detection rate. On the other hand, MTA is not specific to AD, as it has also been observed in cases of AD with cerebrovascular disease and mixed dementia (29).

Few studies from low and middle-income countries have investigated the utility of MTA and the PA scores and their associations with prodromal AD or AD. Similar to our study, one study from China reported that age-adjusted cutoff scores showed better diagnostic accuracy for detecting AD than the non-age-based scores, but was less accurate for distinguishing prodromal AD from controls (33). Another study from China also demonstrated that combining the MTA and the PA had the highest discriminative power for differentiating AD from controls (30). Next, two studies [one from South Korea (34) and one from the Netherlands (35)] also found that the optimal cutoff for axial MTA scores for discriminating AD from healthy controls increased with age, similar to findings from our study (34). Lastly, one study from India also assessed the reliability of using MTA as a visual rating score for detecting AD and found that the MTA scores strongly correlated with cognitive testing results (36). Our findings are consistent with that of other international studies demonstrating the utility of adjusted MTA and MTA+PA scores for distinguishing prodromal AD and AD from cognitively healthy controls.

We found limited studies in Latin America that utilized the MTA and PA scores for comparisons, limiting comparisons with the results of our study in a Peruvian population. One review article from Brazil summarized several articles that have utilized various MRI hippocampal measurements to identify dementia types (37). One study from Argentina used the MTA score to identify patients with AD utilizing the ATN protocol for Alzheimer's disease (38), but this study did not seek to validate the MTA in a Latin American population. Therefore, our study is the first to do so in Latin America.

The majority of prior studies assessing these visual scales are from high-income countries. One study from Norway found that in adjusted models, memory function, APOE4 status, and age were significant predictors of disease progression from prodromal AD to AD, but the MTA scale score was not (39). Our study was cross-sectional and did not assess conversion from prodromal AD to AD, but its clinical utility for these diagnoses has been established by our study. Similar to our study, another study from the United Kingdom found that adding the PA to the MTA scale score improved discrimination of AD from frontotemporal lobar dementia, as well as early-onset AD from normal aging (40).

Other studies have found that when the PA score is applied in isolation to discriminate AD from controls, it has little diagnostic value (33); however, when combined with the MTA score, it can improve its discriminative power (30), as was observed in our study. These findings are consistent with the neurodegeneration patterns that occur as part of the disease process of typical AD cases where pathological changes first occur in the medial temporal lobe and then extend to the posterior cortical regions and in posterior cortical atrophy in atypical variants of AD (40). Moreover, because neurodegeneration predominantly involves the temporal lobes early in the disease course, it would be expected that the MTA score would have higher sensitivity for detection of prodromal AD than the PA score alone. However, since this is a cross-sectional study, we would be unable to determine if posterior atrophy develops in those with prodromal AD (41). As our study has demonstrated, the addition of the PA to the MTA increases sensitivity for the detection of AD. Furthermore, posterior cortical atrophy has been demonstrated in cases of semantic variant of frontotemporal dementia (42), thus combining the MTA and PA scores may be clinically useful for identifying other types of dementia as well (43). As expected, the WMH score does not differentiate the study groups, consistent with prior publications (10, 44). The application of these MRI visual rating scales for the detection of prodromal AD and AD in Latin America, where limited access to CSF and brain PET using amyloid and tau exists may aid the neurologist, geriatrician, or radiologist in the community in identifying patients with these conditions.

In Latin America (LA), only Argentina, Colombia, and Mexico have access to local research laboratories where processing CSF biomarkers, brain PET using amyloid or tau tracers, and glucose metabolism can be performed (2, 5). Other LA countries, such as Peru, only have access to biomarkers through clinical trials relying on CSF sample processing in the United States. Thus, low and middle-income countries require clinical consensus criteria for the timely detection of different stages of AD dementia and prodromal AD through the application of brief cognitive tests, adapted and validated for each region, including evaluation of individuals with low education and urban and rural populations with illiteracy. Once this has been completed, then ancillary tests such as neuroimaging visual rating scale scores can be applied since access to CSF biomarkers in the clinical setting is not readily available in LAC. Moreover, computed tomography is available in all countries, and MRI is available in most Latin American countries, therefore, validating that MRI visual rating scales are particularly needed for Latin America (2, 5).

Our study has limitations. First, our patients did not have pathological confirmation of their diagnoses and there was no follow-up of prodromal AD cases longitudinally to confirm their conversion to AD, highlighting an area of potential investigation in the future. However, AD, prodromal AD cases, and healthy controls were evaluated using CSF amyloid and tau levels known to have high specificity and sensitivity for prodromal AD and AD, improving the diagnostic certainty of these cases. A second limitation is that the study participants were selected from a specialized memory clinic, so those identified as cognitively normal may not be representative of the normal healthy population. Third, we enrolled patients with typical AD and did not enroll those with atypical AD presentation, which could influence the PA scores. A fourth limitation is that the proportion of participants older than 85 years was low and the proportion of participants younger than age 65 was also low, therefore our results should not be extrapolated to these groups of patients. Next, we did not collect data on APOE genotype, posing another limitation to the findings of this study. Lastly, the cut-off scores of the MTA are higher than previously reported, therefore larger-scale community-based studies are needed to assess the predictive value of brain MRI-based visual scales.

## Conclusion

The combination of MTA and PA visual score scales demonstrates greater discriminative ability to differentiate controls from prodromal AD and AD, highlighting the diagnostic value of these visual rating scales as a neuroimaging biomarker in daily clinical practice. A longitudinal follow-up of patients with prodromal AD to determine if they develop AD and the predictive ability of these MRI visual rating scales is needed, particularly for Latin America where adjunct non-invasive biomarkers are needed to confirm the diagnosis of prodromal AD and AD.

## Data availability statement

The raw data supporting the conclusions of this article will be made available by the authors, without undue reservation.

## Ethics statement

The studies involving human participants were reviewed and approved by Hospital Nacional Docente Madre-Niño-HONADOMANI San Bartolomé. The patients/participants provided their written informed consent to participate in this study.

## Author contributions

All authors have participated in the conception and design of the study, data collection and acquisition, writing and critical revision of the article, and approved the final version.

## Funding

This work was self-funded. NC and RM were supported by the National Institute of Health (R56AG069118-01) and Multi-Partner Consortium to Expand Dementia Research in Latin America (ReDLat), supported by the National Institutes of Health, National Institutes of Aging (R01 AG057234). MD was supported by the National Institute of Health (1-K23-MH131466-01), Alzheimer's Association (AARGD-22-924896), and the American Academy of Neurology.

## Conflict of interest

The authors declare that the research was conducted in the absence of any commercial or financial relationships that could be construed as a potential conflict of interest.

## Publisher's note

All claims expressed in this article are solely those of the authors and do not necessarily represent those of their affiliated organizations, or those of the publisher, the editors and the reviewers. Any product that may be evaluated in this article, or claim that may be made by its manufacturer, is not guaranteed or endorsed by the publisher.
